# Editorial: Postharvest disease management in fruits and vegetables: recent advances and mechanisms

**DOI:** 10.3389/fmicb.2023.1203010

**Published:** 2023-05-10

**Authors:** Junfeng Guan, Kaifang Zeng, Zhihui Chen

**Affiliations:** ^1^Institute of Biotechnology and Food Science, Hebei Academy of Agriculture and Forestry Sciences, Shijiazhuang, China; ^2^College of Food Science, Southwest University, Chongqing, China; ^3^College of Life Science, University of Dundee, Dundee, United Kingdom

**Keywords:** postharvest diseases, fruits and vegetables, disease management techniques, biocontrol agents, resistance mechanism

## Introduction

Postharvest diseases can result in significant losses in the quality and economic value of fruits and vegetables during transportation, storage, and marketing. These losses can range from 10 to 50% or more of the total harvest and can have severe consequences for food security and economic stability, especially in developing countries where agriculture is a significant source of income and food supply (Hodges et al., 2011). To prevent or minimize postharvest disease, various management techniques such as sanitation of storage facilities, temperature management, use of resistant varieties, chemical treatments, and biological control are employed (Singh and Sharma, 2018).

Temperature management is crucial in preventing physiological deterioration, moisture loss, and shriveling, as well as reducing the incidence of postharvest diseases. The appropriate temperature can slow the rate of postharvest decay by inhibiting the growth of pathogens, delaying the ripening or senescence of the fresh produce, or both (Singh and Sharma, 2018). The use of low temperatures is an important method to control decay in many fruits and vegetables (Corrales-García and Canche-Canche, 2008; Devanesan et al., 2011). In this Research Topic, Hou et al. conducted a study on the microbial diversity of postharvest Yuluxiang pear (*Pyrus* × *michauxii* “Yuluxiang”) fruits stored at low temperatures. During low-temperature storage, it was found that *Ascomycota* was the dominant fungus at the phylum level, while *Alternaria* was the primary one at the genus level. *Aureobasidium* and *Didymella* were positively correlated with soluble solids and the firmness of fruits, whereas *Phoma* was positively correlated with titratable acid and *Aspergillus* was positively correlated with both titratable acid and firmness.

Climate conditions, such as temperature, humidity, and precipitation, as well as orchard management activities, have been shown to be correlated with the risk of postharvest fungal disease development during fruit storage (Dutot et al., 2013; Bui et al., 2021). To illustrate the impact of climate conditions on postharvest disease management, El-Araby et al. provided an interesting result that shows the relationship between the climate conditions and postharvest microbial load of strawberries in the Gharb and Loukkos regions of Morocco. The study found that climate conditions have a strong influence on postharvest microbial load, with fungal contamination being prevalent in the Gharb region and bacterial contamination being prevalent in the Loukkos region. The authors suggest that postharvest fruit storage must consider the climate of the growing region of strawberries.

Biocontrol methods are considered one of the more sustainable postharvest approaches to extending the shelf-life of fruits and vegetables (Palumbo et al., 2022). *Bacillus* species have been shown to have biocontrol capacity predominantly through inhibitory activity on the growth of plant pathogens, as well as inducing systemic resistance in plants and competing for ecological niches with plant pathogens (Fira et al., 2018; Jinal and Amaresan, 2020). *Bacillus* species are considered to be an eco-friendly and bio-safe alternative to traditional chemical fungicides/bactericides due to their intrinsic ability to induce native anti-stress pathways in plants (Lastochkina et al., 2019). *Bacillus* species are the main promising agents for the biological control of postharvest diseases and have been shown to be effective against pathogens in postharvest fruits (Lastochkina et al., 2019; Wang et al., 2021). Yuan et al. investigated the biocontrol activity and underlying action mechanism of *Bacillus halotolerans* strain Pl7 against *Botryosphaeria dothidea*, the pathogen responsible for apple ring rot. The *B. halotolerans* strain Pl7 possesses cellulase, β-1,3-glucanase, and protease activity and mediates the antifungal activity against *B. dothidea*. *Bacillus halotolerans* strain Pl7 was identified as a promising microbial biocontrol agent against apple postharvest decay due to its ability to swiftly colonize and thrive in surface wounds and change the expression of gene functioning in plant secondary metabolite biosynthesis and plant–pathogen interaction in apple fruit. Ahmad et al. isolated *Bacillus subtilis* strain Y17B from the soil, which exhibited significant antifungal activity against *Alternaria alternata*, the pathogen responsible for fruit rot in cherries. The authors identified surfactin, iturin, and fengycin in the extracted lipopeptide (LP) crude of *B. subtilis* Y17B and found that these LPs were highly effective in reducing the growth of *A. alternata* both *in vitro* and *in vivo*. These results highlight that the biocontrol potential of LPs produced by *B. subtilis* Y17B might be used as an effective biological control agent against *A. alternata* in cherries.

The development and use of resistant genotypes are important means of preventing postharvest diseases in fruits and vegetables (Wilson and Wisniewski, 1989; Singh and Sharma, 2018). Wahengbam et al. investigated the metabolic compounds associated with postharvest physiological deterioration (PPD) progression in resistant and susceptible cassava genotypes. The authors found a significant, strong, and positive correlation between secondary metabolites and gene expression of PPD signaling, which was inversely correlated with hydroxycoumarin and H_2_O_2_ accumulation. MNP Local A tubers, a cassava genotype, exhibited a longer storage life of 15 days with a low PPD score, higher metabolite synthesis, and *PAL* gene expression. These findings suggest that MNP Local A tubers could be a valuable genetic resource for targeted cassava improvement programs aimed at reducing PPD.

## Author contributions

The manuscript was written by JG. The manuscript was reviewed by KZ and ZC. All authors contributed to the article and approved the submitted version.
